# Plasmablastic Richter Transformation of Chronic Lymphocytic Leukemia With Aberrant Epithelial Marker Expression Presenting as a Pleural Effusion: A Diagnostic Challenge

**DOI:** 10.1155/crip/5618071

**Published:** 2026-10-01

**Authors:** Jamie Rogers, Jacky Zahedha Akhter, Marilin Rosa, Sara Niyazi, Dietrich Werner-Idiaquez, Javier Pinilla-Ibarz, Ariel Grajales-Cruz, Lynn Moscinski, Christopher B. Ryder

**Affiliations:** ^1^ Departments of Pathology and Malignant Hematology H. Lee Moffitt Cancer Center Tampa Florida USA moffitt.org; ^2^ Morsani College of Medicine University of South Florida Tampa Florida USA usf.edu

## Abstract

Richter transformation (RT) is a well‐known complication of chronic lymphocytic leukemia (CLL), and it is usually characterized by high‐grade transformation to diffuse large B‐cell lymphoma or, less commonly, to classic Hodgkin lymphoma. Rare reports describe RT presenting as plasmablastic lymphoma or myeloma. In this scenario, complete clinicopathological evaluation is critical to correctly classify the atypical transformation, and demonstration of a clonal relationship with precursor CLL is essential to confirm RT. Herein, we present a case of CLL with plasmablastic RT diagnosed on pleural effusion. The rarity of this phenomenon, compounded by aberrant expression of markers of epithelial differentiation, posed a significant diagnostic challenge. The unusual presentation in our case emphasizes the need for a comprehensive approach integrating clinical, morphologic, phenotypic, and molecular data to avoid misclassification and to guide appropriate clinical management.

## 1. Introduction

Richter transformation (RT) is a recognized aggressive transformation of chronic lymphocytic leukemia (CLL), most often progressing to diffuse large B‐cell lymphoma (DLBCL) and, less commonly, to classic Hodgkin lymphoma (cHL) [1, 2]. RT must also be distinguished from accelerated CLL and pseudo‐RT, the latter of which can be observed upon discontinuation of Bruton tyrosine kinase inhibitor therapy (i.e., ibrutinib) and is reversible with reinitiation of therapy [3, 4]. In exceedingly rare cases, CLL can undergo RT with plasmacytic/plasmablastic differentiation [5–7]. Neoplasms with plasmablastic morphology that enter the differential diagnosis include plasmablastic lymphoma (PBL), plasmablastic myeloma (PBM), and primary effusion lymphoma (PEL), among others, as outlined in the current *WHO Classification of Haematolymphoid Tumors* [8].

Differentiating RT‐PBL from RT‐PBM can be especially challenging, as PBL and PBM are often morphologically and immunophenotypically indistinguishable [2, 8]. Helpful ancillary data such as EBV positivity, HIV and/or HHV‐8 associations, and clinical myeloma‐defining features guide accurate diagnosis [2]. Importantly, confirmation of RT requires evidence of shared clonality between the CLL and transformed populations. This distinction is critical because RT carries a significantly worse prognosis than development of a second, clonally unrelated lymphoma/plasma cell neoplasm [9].

The patient described herein, with a history of CLL on observation, presented with pleural effusion initially reported as containing “highly atypical large cells” with an unusual immunohistochemical profile. The subsequent complete workup illustrates the complexity of diagnosing RT‐PBL, particularly in an effusion‐based specimen.

## 2. Case Presentation

The patient is a 75‐year‐old male with a 5‐year history of treatment‐naïve CLL with unmutated *IGHV* status and Trisomy 12. He presented to an outside institution with significant abdominal pain and imaging findings including bilateral pleural effusions, a hypermetabolic 5.4‐cm mediastinal mass (SUVmax 16.6), and multiple FDG‐avid mesenteric lymph nodes. Because of persistent pleural effusions with anaplastic cells, the patient was referred to our cancer center for further evaluation.

Review of the outside pleural fluid specimen demonstrated large, anaplastic cells of uncertain lineage admixed with background small lymphocytes; immunohistochemical studies were not feasible due to limited cellularity. A repeat pleural fluid specimen obtained at our institution showed large, markedly pleomorphic cells with multinucleation and prominent nucleoli in a lymphocytic background (Figure 1A,B). Given incomplete clinical history at the time of evaluation, a broad immunohistochemistry (IHC) panel was performed. The atypical cells showed positivity for MOC31 (Figure 1C), OSCAR (subset), and variable perinuclear expression of AE1/AE3/CAM5.2 (Figure 1D), whereas hematolymphoid, mesothelial, melanocytic, and neuroendocrine markers were largely negative. Strong CD138 expression prompted hematopathology consultation (Figure 1E).

**Figure 1:**
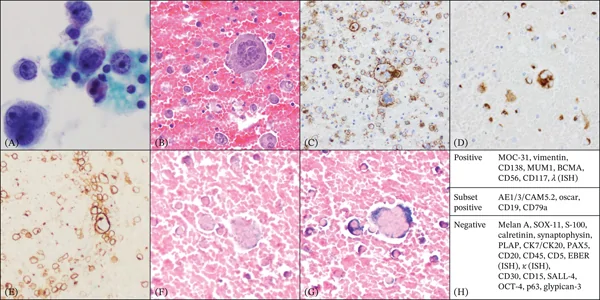
(A) Pleural fluid cytology and (B) corresponding cell block demonstrate scattered large, anaplastic cells with multilobulated nuclei, prominent nucleoli, and abundant cytoplasm with blebbing admixed with smaller cells, some of which show plasmacytic/plasmablastic morphology. The atypical cells on cell block demonstrate expression of (C) MOC31 and (D) AE1/3/CAM5.2 (variable, perinuclear), and (E) CD138 and (F,G) show lambda light chain restriction based on kappa and lambda light chain in situ hybridization (ISH). (H) Summary of immunostaining profile.

Additional IHC and in situ hybridization (ISH) demonstrated lambda light chain restriction (Figure 1F,G) and expression of CD56, BCMA, and vimentin with focal, faint CD19 and CD79a staining by the pleural infiltrate. The plasmablastic cells were negative for viral markers, including Epstein–Barr virus‐encoded RNA (EBER) by ISH and for HHV8/LANA‐1 by IHC. Pleural fluid flow cytometry demonstrated a small population of clonal, CD5‐positive B cells and a more prominent population of plasmacytic cells with shared lambda light chain restriction, suggestive of a B‐cell lymphoproliferative disorder with plasmacytic differentiation. B‐cell receptor (BCR) gene rearrangement studies were not performed on this specimen due to coexisting CLL cells. Serum protein electrophoresis and immunofixation studies remained negative (aside from a later therapeutic daratumumab band). The myeloma fluorescence in situ hybridization (FISH) panel performed on the post‐R‐CHOP (rituximab, cyclophosphamide, doxorubicin, vincristine, and prednisone) bone marrow specimen was also negative.

Based on lymphomatous presentation with extensive pleural fluid involvement and hypermetabolic mediastinal mass, the findings were highly concerning for RT‐PBL, though a plasma cell neoplasm could not be formally excluded—especially given expression of CD56 and CD117 by the plasmacytic cells. The patient was initiated on R‐CHOP chemoimmunotherapy. Restaging positron emission tomography (PET) after cycle one showed decreased metabolic activity of the mediastinal mass, but the patient continued to require thoracentesis after four cycles, resulting in repeat evaluation of pleural fluid. At this point, pleural fluid analysis identified persistent plasmablastic neoplasm with no residual CLL component. BCR gene rearrangement studies identified clonal peaks that matched subsequent results from post R‐CHOP bone marrow biopsy involved only by CLL. This demonstration of shared clonality finally confirmed RT (Table 1). FISH testing on the pleural fluid identified *MYC* rearrangement, gain of Chromosome 1q21, and Trisomy 12, whereas bone marrow karyotype identified only Trisomy 12, further solidifying the clonal relationship and revealing clonal evolution.

**Table 1:** Clonal peak sizes (in bp) of different sampled tissue specimens.

	FR1	FR2	FR3	KA	KB
Pleural fluid	348.28	284.12	161.03	Neg	283.93; 374.73
Bone marrow	348.36	284.09	161.16	Neg	210.92; 283.98; 374.73
PBL	349.30	284.06	161.11	148.92	283.75; 374 (minor)
CLL	348.00	284.03	161.05	148.74	283.88; 374.76

*Note:* FR, Ig heavy chain framework; KA, KB: Ig kappa light chain A and B primer sets.

Vincristine was dose‐reduced during Cycles 4 and 5 because of Grade 3 peripheral neuropathy. PET imaging after five cycles demonstrated a residual, partially calcified anterior mediastinal mass with low‐level FDG uptake (SUVmax 6.1). No further thoracentesis was required, and patient remained stable on observation for 11 months, at which time imaging identified interval increase in adenopathy, including an inguinal lymph node, and PET imaging revealed inguinal lymphadenopathy and a new FDG‐avid right pleural mass (SUVmax 18.9) with fissural nodularity.

Biopsy from the left external iliac lymph node revealed persistent CLL (Figure 2); biopsy of the right pleura demonstrated PBL (Figure 3). BCR gene rearrangement studies performed on both biopsies showed shared clonal peaks matching those previously identified (Table 1). NGS testing performed on RT‐PBL identified *NRAS* G12D mutation.

**Figure 2:**
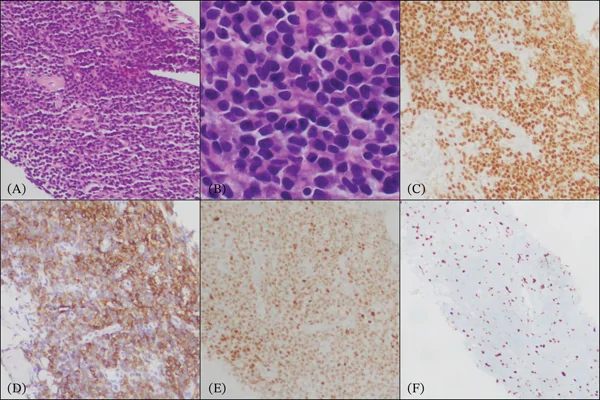
Core biopsy of left external iliac lymph node at (A) low power and (B) high power showing architectural effacement by small, mature lymphoid cells. The tissue is composed predominantly of B‐lineage cells positive for (C) PAX5, (D) CD20 (variable), and (E) LEF1 (dim) with a (F) low Ki‐67 proliferative index of 10%–15%. These cells are also positive for CD23 (not shown).

**Figure 3:**
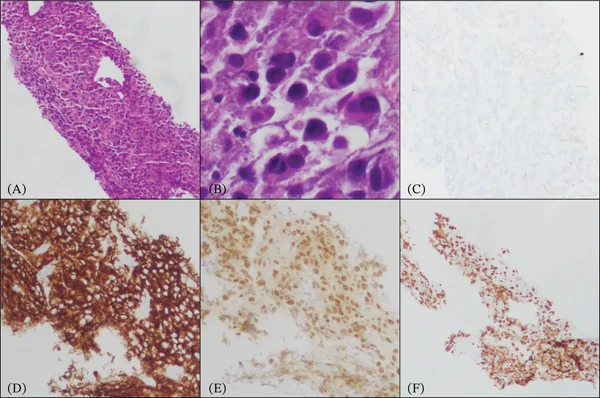
Biopsy of right pleural mass at (A) low and (B) high power demonstrating a proliferation of large, atypical cell with plasmacytic/plasmablastic morphology including vague perinuclear hof and round, eccentric nuclei with prominent nucleoli and occasional multilobation. There is background cellular debris/necrosis. The atypical cells are negative for (C) PAX5 and positive for (D) CD138 and (E) MUM1 with an (F) increased Ki‐67 proliferative index of ~75%. These cells are also negative for CD3, CD20, CD30, EBER (ISH), ALK1, and HHV‐8 (not shown).

Subsequent therapies included zanubrutinib with pembrolizumab (discontinued due to progressive disease) followed by elranatamab and tocilizumab, which were discontinued due to Grades 2–3 cytokine release syndrome. The patient eventually achieved near‐complete metabolic response on daratumumab, lenalidomide, and dexamethasone (DRd). At present, the patient continues DRd therapy (14 months) with sustained unconfirmed complete remission and no recurrent pleural effusions.

## 3. Discussion

RT‐PBL has been rarely reported [6, 7, 10]. Overall, RT occurs in approximately 2% to 10% of CLL cases and consists of histological evolution to aggressive lymphoma, most commonly DLBCL and less often cHL [8, 11–13]. Initiating factors may include immune dysfunction and acquisition of additional genetic changes, such as *MYC* rearrangement (as in this case), that facilitate transformation to high‐grade lymphoma [6, 11]. Careful morphological evaluation and clinical correlation are necessary to differentiate RT from histologically aggressive CLL progression and pseudo‐RT, neither of which carries as negative prognostic implications as true RT.

In our case, where a treatment‐naïve CLL patient presented to our referral center with pleural effusions, mediastinal mass, and abdominal lymphadenopathy, the identification of anaplastic, overtly malignant cells in pleural fluid prompted a broad IHC panel. Initial positivity for multiple epithelial markers and absence of CD45, CD20, and PAX5 raised concern for metastatic carcinoma; however, subsequent organ‐specific epithelial markers were uniformly negative. In addition, the cytomorphology—characterized by singly dispersed cells with eccentric nuclei, prominent nucleoli, and plasmacytoid features—was atypical for carcinoma. Although expressed in various tissues and tumors of epithelial origin, strong CD138 expression prompted hematopathology consultation and additional ancillary studies, which demonstrated shared genetic aberrations and identical clonal BCR gene rearrangements between the patient′s known CLL and the pleural infiltrate, ultimately leading to a diagnosis of RT‐PBL.

The differential diagnosis for a plasmacytoid or anaplastic pleural fluid–based neoplasm is broad and includes PBM, PBL, PEL, ALK‐positive large B‐cell lymphoma, and fluid overload–associated large B‐cell lymphoma, as well as poorly differentiated metastatic carcinoma (Table 2) [8, 14]. Distinguishing among these entities is critical because they differ substantially in prognosis and therapeutic approach. RT‐PBL shows particularly aggressive clinical behavior and inferior survival outcomes compared with conventional CLL and even RT‐DLBCL [5, 8, 9, 11, 14, 15].

**Table 2:** Differential diagnostic considerations for plasmablastic/anaplastic pleural fluid–based neoplasm.

Differential Diagnosis	Morphology	Phenotype	Molecular Features
Plasma cell neoplasm (plasmablastic myeloma)	Plasmablastic cells with minimal perinuclear hof, often admixed with typical, nucleolated plasma cells	CD138+, MUM1+, PAX5−, CD19−, CD56+ (60%–80%+), light chain restriction, keratin− (rarely +), EBV−, HHV‐8−	IgH rearrangement, hyperdiploidy, 1p/1q abnormalities; *MYC* rearrangement rare
Plasmablastic lymphoma	Large plasmablastic cells, high nuclear‐to‐cytoplasmic ratio, vesicular nucleus with prominent nucleoli	CD138+, MUM1+, **PAX5**−, CD19−, CD20−, CD45−/+, EBV+ (60%+), CD56 (20%‐30%+), keratin− (rarely +)	HIV and EBV associated; *MYC* rearrangement common
Primary effusion lymphoma [9]	Immunoblastic, plasmablastic, or anaplastic cells; abundant basophilic cytoplasm +/‐ vacuoles	CD138+, MUM1+, CD20−, PAX5−, CD30+, **HHV-8*+***, EBV+ (80%+), CD45−	HHV‐8 positive; *MYC* rearrangement common
ALK‐positive large B‐cell lymphoma	Immunoblastic to plasmablastic; less often anaplastic	CD138+, CD19−, CD20−, **ALK+**, PAX5 −/+ (20%+), MUM1+, keratin −/+ (15%+), EBV−, HHV‐8−	*ALK* fusion (multiple partners)
Fluid overload‐associated large B‐cell lymphoma	Varied from centroblastic to anaplastic; occasionally plasmacytoid	CD138−, CD20+ (80%+), **PAX5+**, CD30−/+ (25%+), **HHV-8−**	*MYC* rearrangement common
Poorly differentiated metastatic carcinoma	Irregular, pleomorphic cells; clusters, and necrosis common	Variable epithelial markers (AE1/3, CAM5.2, CD138), CD45−, hematologic markers negative	No specific hematologic clonality, variable mutations

*Note:* Bolded phenotypic markers represent key distinguishing characteristics of different entities.

PBL is characterized by loss of conventional B‐cell markers and acquisition of plasma cell–associated antigens [6, 7, 11, 15–17]. According to current WHO frameworks, an essential diagnostic criterion for distinguishing PBL from other entities, such as immunoblastic DLBCL, is the loss of PAX5 expression. Furthermore, strong and diffuse expression of the plasma cell marker CD38 is characteristic (confirmed by flow cytometry in this case) and represents a critical therapeutic target [5, 8].

Molecular studies provided central evidence for RT rather than a second, unrelated plasma cell neoplasm, with PCR‐based BCR clonality analysis demonstrating essentially identical clonal peak sizes (Table 1) and shared Trisomy 12 in both CLL and PBL components. The molecular findings fulfill contemporary criteria for RT. Together with the absence of myeloma‐defining clinical and laboratory features described above, these findings argue against systemic plasma cell myeloma.

Both PBL and PBM are critical diagnostic considerations in RT with plasmacytic/plasmablastic morphology and immunophenotype, as both have been reported in patients with CLL [7, 10, 13]. For example, Castonguay et al. described clonally related progression of CLL to PBM with myeloma‐defining events [12]. The lineage plasticity exhibited by RT reinforces the need for integrated morphologic, immunophenotypic, and molecular evaluation. Unlike the case described by Castonguay et al., our case presented as an extranodal, plasmablastic, mass‐forming lesion without myeloma‐defining features. However, this RT‐PBL did harbor genetic abnormalities associated with myeloma, including 1q21 gain, IgH copy number increase, and an *NRAS* p.G12D mutation. Importantly, these abnormalities are also recurrent in PBL. This illustrates that myeloma‐associated genetic lesions are insufficient for a myeloma diagnosis in isolation; rather, they must be interpreted in the context of morphology, disease distribution, and full clinicopathological workup [2, 8, 9].

The aberrant expression of epithelial markers in this case represents an additional diagnostic pitfall. Although CD138 is expressed by certain epithelial tissues and tumors, conversely aberrant cytokeratin expression can occur in plasma cell neoplasms, PBL, and other lymphomas [18–20]. Awareness of this phenomenon is essential, particularly in cases that exhibit plasmablastic or anaplastic morphology and in effusion‐based specimens, where limited material and incomplete clinical history may predispose to misclassification as carcinoma.

Regarding treatment, previous reports have employed both myeloma‐based and lymphoma‐based regimens for RT‐PBL/PBM, yet outcomes have generally remained poor despite aggressive multimodal therapy [6, 7, 10, 12, 15, 18]. In contrast, our patient′s clinical course is notable for durable survival status after sequential exposure to R‐CHOP, BTK inhibitor‐ and checkpoint inhibitor‐based therapy, a BCMA‐directed bispecific antibody, and ultimately DRd. This relatively prolonged survival compared with previously described cases underscores the potential benefit of integrating novel and targeted therapies for RT‐PBL. Our case, along with a similar RT‐PBL reported by Mazumder et al., specifically suggest the utility of daratumumab‐based therapy for this entity [13].

This case emphasizes the importance of a multidisciplinary approach with disclosure of a complete clinical history to the pathologist, as well as a comprehensive diagnostic approach including morphology, IHC, flow cytometry, and molecular studies. It is also a reminder to not rely on individual IHC marker expression, but rather on a broader panel approach to avoid misdiagnosis.

## Funding

No funding was received for this manuscript.

## Consent

This manuscript was deemed by the authors to not contain potentially identifying information according to International Committee of Medical Journal Editors recommendations. Consent to publish was not explicitly obtained from the patient.

## Conflicts of Interest

The authors declare no conflicts of interest.

## Data Availability

The data that support the findings of this study are available on request from the corresponding author. The data are not publicly available due to privacy or ethical restrictions.
